# Clinical aspects of human mpox cases in the global scenario: an integrative review

**DOI:** 10.15649/cuidarte.3610

**Published:** 2024-11-22

**Authors:** Ana Clara Dantas, Cyntia Leenara Bezerra-da Silva, Dase Luyza Barbosa-de Sousa Alves, Amanda Barbosa-da Silva, Hanna Priscilla da Silva-Medeiros, Allyne Fortes-Vitor

**Affiliations:** 1 Federal University of Rio Grande do Norte, Natal,Brazil . E-mail: anaclaradaantas@yahoo.com.br Federal University of Rio Grande do Norte Natal Brazil anaclaradaantas@yahoo.com.br; 2 Federal University of Rio Grande do Norte, Natal, Brazil. E-mail: cyntialeenara@gmail.com Federal University of Rio Grande do Norte Natal Brazil cyntialeenara@gmail.com; 3 Federal University of Rio Grande do Norte, Natal, Brazil. E-mail: daseufrn@gmail.com Federal University of Rio Grande do Norte Natal Brazil daseufrn@gmail.com; 4 Federal University of Rio Grande do Norte, Natal, Brazil. E-mail: amandaba641@gmail.com Federal University of Rio Grande do Norte Natal Brazil amandaba641@gmail.com; 5 Federal University of Rio Grande do Norte, Natal, Brazil. E-mail: silvahannap@gmail.com Federal University of Rio Grande do Norte Natal Brazil silvahannap@gmail.com; 6 Federal University of Rio Grande do Norte, Natal, Brazil. E-mail: allynefortes@gmail.com Federal University of Rio Grande do Norte Natal Brazil allynefortes@gmail.com

**Keywords:** Monkeypox, Global Health, Disease Prevention, Nursing Care, Review, Viruela del Mono, Salud Global, Prevención de Enfermedades, Atención de Enfermería, Revisión, Varíola dos Macacos, Saúde Global, Prevenção de Doenças, Cuidados de Enfermagem, Revisão

## Abstract

**Introduction:**

In recent years, human-to-human transmission of MPXV has been frequently reported. Despite this, there is a lack of studies with strong evidence to guide the care practice focused on cases of human mpox.

**Objective:**

To analyze scientific evidence in the literature that addresses clinical aspects related to cases of human mpox on the global scenario.

**Materials and Methods:**

Integrative review conducted using five databases: SCOPUS, Web of Science, ScienceDirect, MEDLINE/PubMed and CINAHL. The last date of database access was October 3, 2024. The selection of studies and review report followed the recommendations of the Preferred Reporting Items for Systematic Reviews and Meta-Analyses (PRISMA) guideline.

**Results:**

With a sample of 58 studies, the main findings were aspects related to signs and symptoms, transmission, diagnosis, prevention and care for the multidisciplinary and nursing teams.

**Discussion:**

No studies were found that frequently and specifically addressed the care for the multidisciplinary team and, above all, the nursing team. Therefore, the results ofthis review mayfacilitate the management of patients with MPXV-related infections.

**Conclusions:**

This study promoted the collection of scientific evidence that supports the care for patients with human mpox for the multidisciplinary team and the nursing team, which contributes to prevention, early detection and treatment of MPXV infection and its possible complications.

## Resumen.

**Introducción:**

En los últimos años, la transmisión del virus MPXV entre humanos se ha informado con frecuencia. A pesar de esto, hay escasez de estudios con evidencia sólida que oriente la práctica asistencial centrada en casos de mpox humana.

**Objetivo:**

Analizar la evidencia científica en la literatura que aborda aspectos clínicos relacionados con casos de mpox humana a nivel mundial.

**Materiales y Métodos:**

Revisión integradora, realizada en cinco bases de datos: SCOPUS, Web of Science, ScienceDirect, MEDLINE/PubMed y CINAHL. La última fecha de acceso a las bases de datos fue el 3 de octubre de 2024. La selección de estudios y el informe de la revisión siguieron las recomendaciones de la guía Preferred Reporting Items for Systematic Reviews and Meta-Analyses (PRISMA).

**Resultados:**

Con una muestra de 58 estudios, los principales hallazgos fueron aspectos relacionados con signos y síntomas, transmisión, diagnóstico, prevención y atención del equipo multidisciplinario y del equipo de enfermería.

**Discusión:**

No se encontraron estudios que abordaran con frecuencia y de forma específica el cuidado del equipo multidisciplinario y, sobre todo, del equipo de enfermería.Por lo tanto, los resultados identificadosen estarevisión puedenfacilitar el tratamiento de pacientes con infecciones relacionadas con MPXV.

**Conclusiones:**

Este estudio promovió el levantamiento de evidencia científicaquerespaldala atencióna los pacientes conMPXV humano para la práctica del equipo multidisciplinario y del equipo de enfermería, que contribuyen a la prevención, detección temprana y tratamiento de la infección por el virus MPXV y sus posibles complicaciones.

## Resumo.

**Introdução:**

Nos últimos anos, a transmissão do MPXV entre humanos passou a ser frequentemente relatada. Apesar disso, observa-se uma escassez de estudos com evidências fortes para orientar a prática assistencial voltada aos casos de mpox humana.

**Objetivo:**

Analisar na literatura evidências científicas que abordem aspectos clínicos relacionados aos casos de mpox humana no cenário mundial.

**Materiais e Métodos:**

Revisão integrativa, realizada em cinco bases de dados: SCOPUS, Web of Science, Science Direct, MEDLINE/PubMed e CINAHL. A última data de acesso nas bases foi em 03 de outubro de 2024. A seleção dos estudos e relatório da revisão seguiu as recomendações da diretriz Preferred Reporting Items for Systematic Reviews and Meta-Analyses (PRISMA).

**Resultados:**

Com uma amostra de 58 estudos, os principais achados foram aspectos relacionados aos sinais e sintomas, transmissão, diagnóstico, prevenção e cuidados da equipe multiprofissional e da equipe de enfermagem.

**Discussão:**

Não foram encontrados estudos que abordassem com frequência e especificamente os cuidados da equipe multiprofissional e, sobretudo, da equipe de enfermagem. Logo, os resultados identificados nesta revisão podem facilitar o manejo ao paciente com infecção relacionada ao MPXV.

**Conclusões:**

Este estudo promoveu o levantamento de evidências científicas que fundamentam a assistência ao paciente com mpox humana para a prática da equipe multiprofissional e da equipe da enfermagem, que contribuem para prevenir, detectar precocemente e tratar a infecção do vírus MPXV e suas possíveis complicações.

## Introduction

Human mpox, formerly known as monkeypox, is a zoonotic disease caused by the mpox virus (MPXV). In recent years, human-to-human transmission has become more frequently reported, raising global concern about its potential for spreading^1^^, ^^4^.

The first case of human mpox in the most recent multinational outbreak was confirmed in the UK on May 6, 2022, in a man travelling from Nigeria. New cases were quickly detected in several other countries, leading the World Health Organization (WHO) to declare a Public Health Emergency of International Concern (PHEIC) on July 23, 2022^5^^, ^^6^.

According to the latest report published on September 28, 2024, a total of 106,310 confirmed cases of human mpox had been reported in 123 locations worldwide, including 234 deaths. The highest number of cases was reported in the United States of America (USA) (33,812 cases), followed by Brazil (12,206 cases) and Spain (8,240 cases) ^7^.

Despite its current relevance, there is a lack of studies with strong evidence to guide care practice focused on cases of human mpox. This study is justified by the importance of compiling information that supports evidence-based practice on the clinical aspects of human mpox.

This study aims to analyze scientific evidence in the literature that addresses clinical aspects related to cases of human mpox in the global scenario.

## Materials and Methods

### Design

This is an integrative review conducted through the following stages: formulation of the research question, identification of relevant studies, selection of studies and extraction of data and, finally, presentation of a synthesis of the evidence^8^^, ^^9^. This study followed the recommendations of the Preferred Reporting Items for Systematic Reviews and Meta-Analyses (PRISMA) ^10^ guidelines. The dataset of this study was stored in the Mendeley Data^11^ public repository.

### Formulation of the research question

The research question was formulated based on the PICO strategy (P: population - general population; I: interest - clinical aspects related to human mpox cases; C: context - world scenario). Thus, the following guiding question was delimited: What is the existing scientific evidence on the clinical aspects related to human mpox cases in the global scenario?

### Identification of relevant studies

Five databases were accessed: SCOPUS, Web of Science, ScienceDirect, MEDLINE and PubMed from the National Library of Medicine and the National Institutes of Health, and Cumulative Index to Nursing and Allied Health Literature (CINAHL). The last database access was on October 3, 2024.

Regarding the inclusion criteria, we considered studies that addressed evidence on cases of human mpox in the world scenario and available in full text. In order to achieve a comprehensive survey of the literature and considering that human mpox is an old infection, whose repercussions are the focus of study worldwide, no time frame was set for publications. Editorials, letters to the editor, abstracts, case reports, case series, experience reports, non-systematic reviews and reflection articles were excluded.

For the search strategy, the descriptors indexed in the Medical Subject Headings (MeSH) were used: "Monkeypox," "Monkeypox virus" "Delivery of Health Care," "Epidemiology," and "Signs and Symptoms". Three search strategies were developed for the SCOPUS database, and were adapted to the other databases, considering their particularities (Table 1).

**Table 1:** Search strategies developed for SCOPUS database

Search strategies
ALL(“Monkeypox" OR “Monkeypox virus”) AND (“Delivery of Health Care")
ALL(“Monkeypox” OR “Monkeypox virus”) AND (“Epidemiology”)
ALL (“Monkeypox" OR “Monkeypox virus") AND (“Signs and Symptoms")

### Selection of studies and data extraction

The search was performed independently by two researchers, finding the same number of studies in each database. The publications found were stored and exported to the software Rayyan - Intelligent Systematic Review (https://rayyan.ai/). Duplicates were removed and studies were selected. The organization of the citations and reference list of this review were managed by the Mendeley reference management software.

After this process, the title and abstract of the studies found were read to analyze whether they answered ourguidingresearch question.The articlesincluded after theinitial readingweresubmitted for full reading. In order to reduce possible biases, two researchers independently performed a selection stage of studies for analysis, with a third researcher to resolve any discrepancies.

For data mapping and data extraction procedure, a structured tool was developed in Microsoft Excel 2019®, containing the following items: identification of studies, methodological aspects, items related to the clinical aspects of human mpox cases, and main conclusions of the studies.

### Synthesis of evidence

An analytical framework was built for data analysis by compiling selected studies, allowing for gathering and synthesizing the main information of the articles. The results were analyzed and later synthesized descriptively with the characterization of the studies.

Regarding the level of evidence for analyzing the studies, we chose to follow the JBI guidelines, which present five levels of evidence, namely: level 1 - experimental studies; level 2 - quasi experimental studies; level 3 - analytical observational studies; level 4 - descriptive observational studies; and level 5 - expert opinions^12^.

## Results

**Figure 1:**
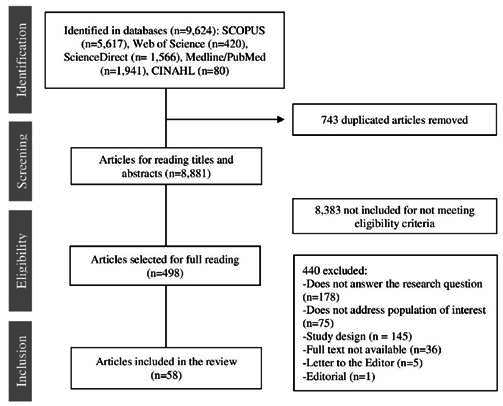
Study selection diagram, according to PRISMA flowchart

The database search stage yielded a total of 9,624 identified studies. After the selection process, 58 studies composed the final sample, as outlined in the flowchart shown in Figure 1.

Most studies were published in 2023, representing 60.34% (35) of the sample; 34.48% (20) were from Europe; 34.48% (20) had a methodological design characterized by systematic review; and 100% (56) were written in English. Table 2 shows the characteristics of the studies included in the sample detailing authors, year of publication, country, journal of publication, study design, and level of evidence. The main findings are presented in Table 3.

**Table 2:** Characterization of selected studies

Authors (year)	Country	Journal	Study design/LE
Ramirez-Soto et al. (2024)^13^	Peru	Journal of Infection and Public Health	Observational study/3.e.
Ribeiro et al. (2024) ^14^	Brazil	Epidemiology and Health Services.	Descriptive study/4.b
Malone et al.(2023) ^15^	USA	International Journal of Environmental Research and Public Health.	Systematic review, with meta- analysis/1.b
Heukelom et al. (2023) ^16^	The Netherlands	Journal of the European Academy of Dermatology and Venereology	Quasi-experimental study/2.c
Wieder-Feinsod et al. (2023) ^17^	Israel	International Journal of Infectious Diseases.	Retrospective Study/2.d
Passini et al. (2023) ^18^	Italy	Pathogens	Retrospective Study/2.d
Assiri et al. (2023) ^19^	Saudi Arabia	Journal of Infection and Public Health	Observational study/2.d
Maldonado et al. (2023) ^20^	Peru	International Society for Infectious Diseases	Observational study/2.d
Brosnan et al. (2023) ^21^	USA	Emerging Infectious Diseases	Observational study/2.d
Núñez et al. (2023) ^22^	Mexico	The Lancet Regional Health.	Observational study/2.d
Deb et al. (2023) ^23^	India	Journal Pre-proof	Systematic Review/3.a
Abdelaal et al. (2023) ^24^	Egypt	Asia-Pacific Journal of Ophthalmology	Systematic review and meta- analysis/3.b
Zaqout et al. (2023) ^25^	Qatar	Journal of Infection and Public Health	Cohort study/3.c
Fink et al. (2023) ^26^	United Kingdom	The Lancet infectious diseases.	Retrospective cohort study/3.c
Sobral-Costas et al. (2023) ^27^	Spain	Journal of the American Academy of Dermatology	Prospective cohort study/3.c
Mazzotta et al. (2023) ^28^	Italy	Journal of Medical Virology	Prospective cohort study/3.c
Morales et al. (2023) ^29^	Spain	Eurosurveillance	Prospective cohort study/3.c
Zucker et al. (2023) ^30^	Israel	Clinical Microbiology and Infection	Retrospective cohort study/3.c
Herrán-Arita et al. (2023) ^31^	Mexico	Microorganisms	Case-control study/3.d
Rimmer et al. (2023) ^32^	United Kingdom	International Journal of Infectious Diseases.	Case-control study/3.d
Sahin Y, et al. (2023) ^33^	Türkiye	Annals of the Brazilian Academy of Sciences	Systematic Review/4.a
Chenchula et al. (2023) ^34^	India	Archives of Virology.	Systematic Review/4.a
Hatami et al. (2023) ^35^	Iran	Biomedicines.	Systematic review, with meta- analysis/4.a
Jaffer et al. (2023) ^36^	USA	American Journal of Otolaryngology	Systematic review, with meta- analysis/4.a
Sharma et al. (2023) ^37^	India	International Journal of Emergency Medicine	Systematic Review/4.a
Du et al. (2023) ^38^	China	International Journal of Public Health	Systematic Review/4.a
Simadibrata et al. (2023) ^39^	Indonesia	Journal of Medical Virology	Systematic Review/4.a
Jaiswal et al. (2023) ^40^	USA	Current Problems in Cardiology	Systematic Review/4.a
Pourriyahi et al. (2023) ^41^	Iran	Journal of Medical Virology	Systematic Review/4.a
Liu et al. (2023) ^42^	China	Pathogens	Systematic review, with meta- analysis/4.a
Velázquez-Cervantes et al. (2023) ^43^	Mexico	Clinical Journal Spanish	Systematic Review/4.a
Khan et al. (2023) ^44^	Nepal	Medicine	Systematic Review/4.a
Gandhi P A, et al. (2023) ^45^	India	EClinicalMedicine.	Systematic Review/4.a
Chaudhari S, et al. (2023) ^46^	USA	Cureus	Systematic Review/4.a
Li et al. (2023) ^47^	China	Signal Transduction and Targeted Therapy	Cross-sectional study/4.b
Angelo et al. (2023) ^48^	Multicenter	The Lancet infectious diseases.	Cross-sectional study/4.b
Eser-Karlidag et al. (2023) ^49^	Multicenter	New Microbes and New Infections	Cross-sectional study/4.b
Webb et al. (2022) ^50^	United Kingdom	BMJ Global Health	Systematic Review/2.a
Nórz et al. (2022) ^51^	Germany	Eurosurveillance	Experimental trial/2.c
Hoffmann et al. (2022) ^52^	Germany	HIV Medicine	Retrospective Study/2.d
Silva et al. (2022) ^53^	Brazil	The Lancet Regional Health.	Prospective cohort study/3.c
Tarín-Vicente et al. (2022) ^54^	Spain	The Lancet	Cohort study/3.c
Acevedo et al. (2022) ^55^	Chile	SSRN eLibrary	Case- control study/3.d
Mailhe M et al. (2022) ^56^	France	Clinical Microbiology and Infection	Observational study/3.e
García-Hernández L, et al. (2022) ^57^	Spain	Spanish Journal of Public Health	Retrospective Study/3.e
Bunge et al. (2022) ^58^	USA	PLOS Neglected Tropical Diseases	Systematic Review/4.a
Jairoun et al. (2022) ^59^	United Arab Emirates	Journal of Infection and Public Health.	Cross-sectional study/4.b
Amao et al. (2022) ^60^	Nigeria	Journal of Public Health	Cross-sectional study/4.b
Martínez et al. (2022) ^61^	Spain	Eurosurveillance	Cross-sectional study/4.b
Antinori et al. (2022) ^62^	Italy	Eurosurveillance	Cross-sectional study/4.b
Peiró-Mestres et al. (2022) ^63^	Spain	Eurosurveillance	Cross-sectional study/4.b
Perez Duque et al. (2022) ^64^	Portugal	Eurosurveillance	Cross-sectional study/4.b
Ricco et al. (2022) ^65^	Italy	Tropical Medicine and Infectious Disease	Cross-sectional study/4.b
Beer et al. (2019) ^66^	United Kingdom	Plos Neglected Tropical Diseases	Systematic Review/2.a
Li et al. (2017) ^67^	USA	The American Journal of Tropical Medicine and Hygiene	Experimental study/2.b
Nolen et al. (2015) ^68^	Democratic Republic of the Congo	The American Journal of Tropical Medicine and Hygiene	Retrospective cohort study/3.c
Formenty et al. (2010) ^69^	USA	Centers for Disease Control and Prevention (CDC)	Retrospective Study/4.b
Huhn et al. (2005) ^70^	USA	Clinical Infectious Diseases	Case-control study/3.d

*Level of evidence according to global JBI^12^

**Table 3:** Main findings regarding the aspects of human mpox cases

Aspects	Main findings
Signs and symptoms	•The incubation period of the MPXV is from 4 to 21 days, with a mean period of 8.5 days^34^^,^^37^^, ^^49^^, ^^56^. •Symptoms such as fever, lymphadenopathy, proctitis, oral and/or anogenital lesions, rash, headache, asthenia, myalgia, chills, malaise, fatigue and ocular complications were presented^14^^, ^^51^^, ^^60^^, ^^61^, while odynophagia and cough are less common^20^. •Rashes begin in the macular phase and then progress to the papular, vesicular, pustular, and crustal phases ^33^^, ^^41^^, ^^51^^, ^^56^
Transmission	•It occurs mostly in males, ranging from 18 to 67 years of age, with a mean age of 35 years^19^^, ^^20^^, ^^25^^, ^^55^. •Human-to-human transmission: associated through respiratory droplets, contact with bodily fluids, environment or belongings of a contaminated patient, skin lesion of an infected person and with sexual transmission of an infected individual with lesions in the groin and genitals^35^^, ^^58^. •Animal-human transmission: direct contact with natural viral hosts or consumption of hosts, or direct contact with blood of an infected animal, body fluids or inoculation through skin mucus lesions^35^^, ^^58^. •The virus is particularly dangerous for pregnant women, children under eight years of age, and people with compromised immune systems^13^^, ^^43^^, ^^66^.
Diagnosis	•Diagnosis is preferably by reverse transcription followed by polymerase chain reaction (RT-PCR) ^19^^, ^^52^^, ^^53^^, ^^56^^, ^^61^. •Optimal diagnostic samples come from rash, fluid, scabs or, in some cases, a biopsy of the lesions^46^. •Inquire about travel and sexual history or any close contact with people exhibiting a similar rash or with suspected or confirmed varicella infection^19^^, ^^38^^, ^^41^. •Samples can also be tested for varicella-zoster virus (VZV) ^41^.
Prevention	•There are no vaccines specifically designed to protect against infection and disease of human mpox^20^. •The smallpox vaccines ACAM-2000 and MVA-BN are prophylactically recommended for people at risk of MPXV exposure^33^^, ^^41^. •The use of the smallpox vaccine Imvanex is recommended for prevention after close contact or exposure to patient with the virus^33^. •For all cases, including suspects, recommendations are made for home isolation under clinical and epidemiological surveillance, use of surgical masks when sharing the same location and avoiding contact with domestic animals. These measures should be maintained until the skin lesions become crusty and all crusts fall off^21^^, ^^45^^, ^^57^. •Healthcare professionals must wear specific Personal Protective Equipment (PPE) for droplet and contact isolation^62^. •Contact screening to control the spread of mpox should be done^61^.
Treatment	•There is no clinically proven specific therapy for mpox disease^37^^, ^^62^. •Treatment is primarily for symptomatic control^56^^, ^^62^. For skin lesions, topical cidofovir is a potentially relevant therapy, but with mild systemic involvement^25^^, ^^27^. •For severe cases, especially in pregnant patients, children under eight years of age or immunocompromised individuals, the use of antiviral drugs to treat smallpox was approved, such as Tecovirimat (TPOXX) ^21^^, ^^25^^, ^^33^.
Care for the multidisciplinary team and the nursing team	•Recognize early cases of MPXV infection through appropriate alert screening, isolation, and infection control protocols^21^^, ^^45^^, ^^57^. •Carry out case screening through active case-finding in health units^20^^, ^^21^. •Monitor possible symptoms such as fever, chills, rash and lymphadenopathy for 21 days after the last exposure^37^^, ^^49^^, ^^56^. •Treat symptoms according to the needs of each patient^56^^, ^^62^. •Provide support to maintain adequate water balance^37^. •Offer hemodynamic support, such as supplemental oxygen or other respiratory support, if necessary^37^. •Treat secondary bacterial infections of skin lesions, when indicated^26^. •Identify and treat eye infections and complications resulting from MPXV infection^34^^, ^^56^. •Offer psychosocial support to the patient and family^60^.

## Discussion

### Signs and symptoms

Regarding the MPXV incubation period, that is, the interval from infection to the onset of symptoms, most studies reported a period of 4 to 21 days, with a mean period of 8.5 days^34^^, ^^37^^, ^^49^^, ^^55^. Before the 2022 outbreak, the mean incubation period of the infection was 5 to 13 days^70^.

Patients typically present with symptoms such as fever, lymphadenopathy, proctitis, oral and/ or anogenital lesions, rash, headache, asthenia, myalgia, chills, malaise, fatigue and ocular complications^51^^, ^^60^^, ^^61^, while odynophagia and cough are less common^20^. Systemic symptoms are followed by the development of characteristic rashes. Generally, the rashes appear in the mouth and soon spread to the face and extremities, involving the palms of the hands and soles of the feet45. Lesions in the anogenital region, present in the anus, perianal region, scrotum and penis, were also reported in the studies^16^^, ^^37^^, ^^42^.

Due to the systemic symptoms that occur initially, resembling those of flu-like syndrome, the initial diagnosis may be ignored. With the manifestation of rashes near the genitals, MPXV is often diagnosed as another Sexually Transmitted Infection (STI) ^70^. There is a need to identify and monitor symptoms for differential diagnosis by health professionals.

Furthermore, the findings show the possibility of complications resulting from MPXV infection, such as subsequent skin infections, pneumonia, sepsis, encephalitis and corneal infection leading to potential vision loss^37^^, ^^46^. Immunocompromised individuals, pregnant women and children under eight years of age are more likely to have severe complications of the disease, with mortality rates ranging from 1 to 11%^66^.

### Transmission

The MPXV can be transmitted from person to person, animal to animal, and animal to human. Human- to-human transmission has been associated with respiratory droplet transmission, contact with bodily fluids, contact with skin lesions of an infected person, and sexual transmission of an infected individual with groin and genital lesions. Furthermore, studies indicate that the environment or belongings of a contaminated patient, such as clothing and bed/bath linens, are capable of transmitting the virus^35^^, ^^58^.

An interesting finding in this study was the transplacental passage of the virus, which may cross the placenta of the infected mother and contaminate the fetus. According to the guidelines, it is recommended that the monitoring of infected pregnant women include fetal monitoring through ultrasound in order to detect possible abnormalities, such as fetal hepatomegaly or dropsy^43^.

Most studies indicate that males are the most frequently reported cases. In men, the observed age was between 20 and 67 years old, with a mean age of 35 years^19^^, ^^20^^, ^^25^^, ^^55^. In the current mpox outbreak, men often self-identify as men who have sex with men, homosexuals and bisexuals^32^^, ^^48^. Research confirms this finding, and this population requires greater attention^71^^, ^^72^.

Another relevant finding relates to the frequent report of the occurrence of MPXV in patients with human immunodeficiency virus (HIV) ^48^. This may be related to the incidence rate in men, who are the main group affected by HIV infection.

A study points out that in relation to exposure rates, heterosexual men account for 49% of cases and homosexuals 38%. In specific populations, such as men who have sex with men, studies indicate a high prevalence of HIV, estimated at about 18.4%^73^. In addition, risk factors were identified among young men, including engaging in risky behaviors and activities, such as condomless sex and being HIV positive^48^^, ^^74^.

### Diagnosis

The diagnosis of MPXV infection was identified in the studies, preferably by reverse transcription followed by polymerase chain reaction (RT-PCR). Samples for RT-PCR tests are taken from blood, throat, anal region, skin vesicles and urine^19^^, ^^52^^, ^^53^^, ^^56^^, ^^61^. However, optimal diagnostic samples come from rash, fluids, crusts, or, in some cases, a biopsy of the lesions^46^.

When clinically suspecting an MPXV infection, health professionals should inquire about the patient’s travel and sexual history, as well as any close contact with people exhibiting a similar rash or with suspected or confirmed virus infection^19^^, ^^38^^, ^^41^.

### Prevention

There are no vaccines specifically designed to protect against MPXV infection. However, numerous studies report that prior smallpox vaccination provides protection against human mpox and mitigates the severity of its clinical presentation, even though vaccine-induced immunity has progressively declined in older individuals^20^^, ^^33^^, ^^41^. The smallpox vaccine is estimated to provide about 85% efficacy against human mpox^66^.

For all cases, including suspected and confirmed contaminated patients, control measures should be implemented for patients and their contacts. It is recommended that patients be isolated at home and that surgical masks be worn by the affected patient and by the contact when sharing the same room. Personal objects, including clothes, towels and sheets should not be shared, and contact with domestic animals should be avoided. These measures should be maintained until the skin lesions have crusted over and all scabs have fallen off^21^^, ^^45^^, ^^57^^, ^^61^.

Healthcare workers should wear specific Personal Protective Equipment (PPE) for droplet and contact isolation, such as an apron, gloves, eye protection and N95/PFF2 masks^62^.

### Treatment

The findings indicate that there is no clinically proven specific therapy for mpox. Treatment is based on symptoms and necessary clinical support, including the use of antipyretics and analgesics, maintenance of hydroelectrolytic balance, nutrition, early identification of secondary infections with prompt treatment with available antimicrobial agents^37^^, ^^62^.

A medication called Tecovirimat, also known as TPOXX or ST-246, was approved for MPXV infection in early 2022. It is recommended for use in severe cases, such as pregnant women and children^21^^, ^^25^^, ^^33^. However, it is not yet widely available and further studies are needed to prove its safety and efficacy.

For skin lesions, topical cidofovir at 1% has been identified as a potentially relevant therapy, but with mild systemic involvement. Its use should be evaluated based on a risk-benefit analysis^25^^, ^^27^.

### Care for the multidisciplinary team and the nursing team

No studies were found that frequently and specifically addressed the care for the multidisciplinary team and, above all, the nursing team. Therefore, the results identified in this review may facilitate the management of patients with MPXV-related infection.

Strategies such as early recognition cases of MPXV infection cases through appropriate alert screening, isolation, and infection control protocols, and active case-finding in health facilities are effective in preventing and controlling widespread outbreaks and should be developed by health professionals. Additionally, possible symptoms such as fever, chills, rash, and lymphadenopathy should be monitored for a period of 21 days after the last exposure to a patient with confirmed infection or cases with characteristic rashes^49^^, ^^56^^, ^^61^.

The interventions adopted by the team that provides care to patients with MPXV infection should be based mainly on treating symptoms, offering support to maintain adequate fluid balance(due to the possibility of increased insensitive fluid losses through the skin, decreased oral intake and vomiting or diarrhea) providing hemodynamic support, such as supplemental oxygen or other respiratory support, if necessary, and treating secondary bacterial infections of skin lesions when indicated^26^^, ^^37^^, ^^56^^, ^^62^.

An important finding is related to management of eye infections and complications, since MPXV infection can specifically result in corneal scarring and/or vision loss. Approaches such as early ocular evaluation, application of lubricants, topical antibiotics, and possibly topical antivirals, such as trifluridine, can be used to avoid potential risks to the patient's vision^35^^, ^^56^. In this context, nursing plays a fundamental role in providing direct care to patients, in order to avoid serious injuries and maintain ocular integrity.

Psychosocial support has also been reported in studies, where professionals must provide assistance according to the needs of the patient and family. Active listening and the transmission of supportive messages can be considered strategies for psychosocial support^60^. Because mpox is a relatively current infection, with an increase in cases occurring in the last year, people may feel threatened and afraid of it, mainly due to the lack of information. Thus, psychosocial support is an intervention considered relevant for mpox patients care.

Thus, it is the responsibility of health professionals, especially the nursing team, who maintain closer contact with the patients, to establish interventions that promote progression of the patient's condition while considering their human responses resulting from infection with the MPXV.

This study has limitations that may relate to the databases selected identifying the studies, which may have contributed to concealing another relevant research on the topic. Regarding the level of evidence, most studies presented a low level, probably because this is a relatively current subject that has been studied little.

It is recommended that scientific studies with stronger levels of evidence, such as experimental or quasi-experimental studies, be developed to provide safer evidence for the care of these patients. It is important to note that the information contained in the results of this study may change as new studies on the subject are conducted.

## Conclusion

Information available in the literature about the main clinical aspects related to human mpox cases was identified, namely: signs and symptoms, transmission, diagnosis, prevention, treatment and care for multidisciplinary team and the nursing team. Based on this, an analysis was conducted, and relevant information was compiled and organized in the results of this review.
